# Return to play and recovery metrics after arthroscopic Bankart repair with augmentations in rugby players

**DOI:** 10.1016/j.xrrt.2025.100649

**Published:** 2025-12-29

**Authors:** Yuki Miyasaka, Shota Hoshika, Keisuke Matsuki, Takashi Takamura, Hideki Kamijo, Tomoyuki Matsuba, Tomoshige Tamaki, Norimasa Takahashi, Hiroyuki Sugaya

**Affiliations:** a Department of Rehabilitation, Funabashi Orthopaedic Clinic, Funabashi, Chiba, Japan; b Sports Medicine and Joint Center, Funabashi Orthopaedic Hospital, Funabashi, Chiba, Japan; c Tokyo Sports & Orthopaedic Clinic, Tokyo, Japan

**Keywords:** Shoulder dislocation, Rugby, Return to sports, Contact, Range of motion, Rehabilitation

## Abstract

**Background:**

There is limited data on the relationship between recovery metrics, including psychological factors, and shoulder function in rugby players after arthroscopic Bankart repair. The purpose of this study was to investigate return to play (RTP) in rugby players who underwent shoulder stabilization for anterior shoulder instability and to assess the relationship between postoperative ranges of motion (ROMs) and various recovery metrics, including performance, pain, and psychological conditions.

**Methods:**

We retrospectively investigated subjects who underwent arthroscopic shoulder stabilization at our institute from January 2012 to April 2022. Inclusion criteria were as follows: (1) rugby players with traumatic anterior shoulder instability and (2) arthroscopic Bankart repair with rotator interval closure and Hill–Sachs remplissage. Exclusion criteria were as follows: (1) revision surgery and (2) incomplete questionnaire at the final follow-up. A questionnaire at the final follow-up was used to evaluate the recovery metrics as a visual analog scale (VAS), including athletic performance, pain, and fear of contact. Regression analyses were performed to assess the relationship between the VAS scores and ROMs.

**Results:**

Ninety-one shoulders in 82 patients met the inclusion criteria, and 32 shoulders were excluded due to revision surgery, one shoulder; incomplete questionnaire, 31 shoulders. The remaining 59 shoulders in 50 patients were included in this study. The patients consisted of 48 males and 2 females, with a mean age at surgery of 19 years (range, 14-36). The mean follow-up was 31 months (range, 13-56). The mean time to start contact practice was 7 ± 5 months (range, 3-36), and the mean time to RTP was 8 ± 5 months (range, 4-36). Forty-nine patients (98%) returned to their preinjury sports level. The median of VAS values were as follows: 91 for athletic performance (range, 25-100), 100 for pain (range, 10-100), and 70 for fear of contact (range, 10-100). ROM data were available in 52 shoulders. Multiple regression analyses showed significant relationships between total VAS scores and ROMs at postoperative three months (anterior elevation, *P* = .015; external rotation at the side, *P* = .002).

**Conclusion:**

Arthroscopic shoulder stabilization resulted in a 98% RTP rate in rugby players. The VAS score for fear of contact was relatively low, even in patients with complete return. The total VAS value was correlated with ROMs at three months postoperatively. A rehabilitation protocol that simultaneously addresses psychological and physical aspects may be required for better RTP.

Anterior shoulder instability is a common injury among contact and collision athletes, such as rugby players, which can impact their athletic careers.^15^^,^^18^^,^^3^^,^^34^ To restore shoulder stability and facilitate return to play (RTP), surgical interventions are often necessary. One commonly employed procedure is arthroscopic Bankart repair, which can effectively restore shoulder stability and is associated with a low recurrence rate.^2^^,^^5^^,^^38^

Many authors have reported high RTP rates among rugby players within 5-7 months following shoulder stabilization surgery.^10^^,^^14^^,^^20^^,^^22^^,^^24^^,^^26^^,^^29^^,^^38^ Since 2012, we have been performing arthroscopic Bankart repair with selective augmentations, including rotator interval closure and Hill–Sachs remplissage for competitive contact/collision athletes with shoulder instability, based on recurrence risk.^10^ Our treatment strategy has yielded satisfactory outcomes with low recurrence and high RTP rates^10^; however, some patients, even those who underwent Hill–Sachs remplissage, have reported anxiety of contact play after RTP. While several metrics, including pathological conditions, psychological factors, and physical characteristics, have been indicated as influencing RTP, limited data exist on the association between recovery metrics and shoulder function, such as ranges of motion (ROMs), in rugby players after arthroscopic Bankart repair.^4^^,^^25^^,^^29^

The purpose of this study was to investigate RTP in rugby players who underwent shoulder stabilization for anterior shoulder instability and to assess the relationship between postoperative ROMs and various recovery metrics, including performance, pain, and psychological conditions. We hypothesized that there would be a relationship between postoperative ROMs and the recovery metrics.

## Materials and methods

This study is a retrospective case study investigating the recovery metrics in rugby players who underwent arthroscopic shoulder stabilization. This study was conducted at a single sports medicine center specializing in shoulder and elbow and was approved by the ethics review board of our institute.

### Patient selection

We retrospectively investigated subjects who underwent arthroscopic shoulder stabilization at our institute from January 2012 to April 2022. Inclusion criteria were as follows: (1) rugby players with traumatic anterior shoulder instability and (2) arthroscopic Bankart repair with rotator interval closure and Hill–Sachs remplissage. Exclusion criteria were as follows: (1) revision surgery and (2) incomplete questionnaire at the final follow-up. Medical records were reviewed to collect patient data, such as gender, age at surgery, injured side, time from initial dislocation to surgery, number of preoperative dislocations/subluxations, athletic level, and playing position in rugby (forwards or backs).

### Surgical procedures

All surgeries were performed by one of 5 shoulder surgeons. In all shoulders, arthroscopic Bankart repair was performed in the beach chair position under general anesthesia.^10^^,^^28^ First, diagnostic arthroscopy was performed throughout the glenohumeral joint. Then, the labroligamentous complex was separated from the glenoid neck, starting from the 2 o'clock position to the 7 o'clock or 7:30 position (in the right shoulder). In addition, a small amount of articular cartilage at the anteroinferior glenoid face was removed to promote tissue healing. The labroligamentous complex was fixed using at least 4 suture anchors while cranially pulling up the complex with a grasper. In shoulders with a bony Bankart lesion, the complex was repaired without resecting the fragment.^17^^,^^27^ Other pathological lesions, such as superior labrum anterior and posterior lesion, capsular tear, humeral avulsion of the glenohumeral ligament lesion, and rotator cuff tear, were repaired as necessary. In addition, rotator interval repair and Hill–Sachs remplissage were added to all shoulders as reinforcement techniques.^10^^,^^28^

### Postoperative protocol

After 3 to four weeks of immobilization in a sling, passive and assisted active exercises were initiated while avoiding pain provocation. Until 12 weeks after surgery, medical rehabilitation primarily focused on ROM recovery. Strength training on the unaffected shoulder and aerobic exercise were also performed. Twelve weeks after surgery, strength training of the affected shoulder was initiated. According to each patient's functional recovery, contact practice was allowed with nonpersonal contact movements sixteen weeks after surgery, such as tackling dummies and practicing with stationary opponents; gradually, they progressed to practicing with responsive opponents. Full RTP was permitted 20-24 weeks after surgery.

### Patient assessment

**Figure 1:**
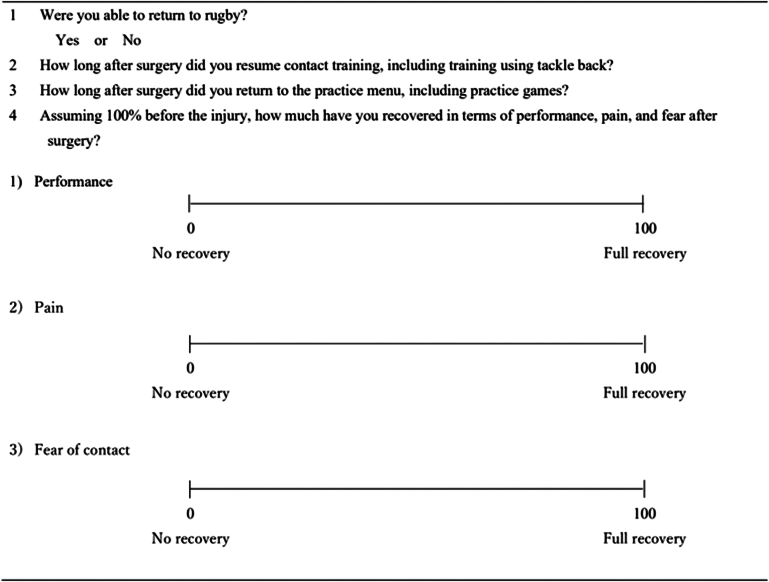
Detail of questionnaire survey.

We used a questionnaire at the final follow-up to evaluate recovery metrics, including athletic performance, pain, and fear of contact (Fig. 1). Patients rated each factor using a 0-100 visual analog scale (VAS), where a score of 100 meant the best condition before injury. The sum of the 3 VAS values was used to analyze relationships with ROMs. Patients were also asked to report the time at which they resumed contact practice and practice games. Contact practice was defined as nonpersonal contact against tackle dummies or contact with stationary opponents. RTP was defined as a complete return to a game. Patients with both shoulder involvement answered the questionnaire for each shoulder.

ROM data were obtained from the medical records. ROMs were assessed using a goniometer in all patients. Anterior elevation (AE), external rotation with the arm at the side (ER1), and external rotation in 90-degree abduction (ER2) were evaluated before surgery and at 3, 6, and 12 months postoperatively.^2^

### Statistical analysis

Single regression analysis was performed with the sum of the athletic performance, pain, and fear of contact VAS values as an objective variable and ROM at each period as an explanatory variable. Multiple regression analysis was conducted to investigate the influence of each ROM on the total VAS values. ROM at each period was used as an explanatory variable. To control for potential confounding variables, follow-up term, age at surgery, time from initial dislocation to surgery, and number of preoperative dislocations/subluxations were also included in the analysis.^30^^,^^35^ A post hoc power analysis was performed based on the results of the regression analysis. R version 4.2.2 (CRAN, Vienna, Austria) was used for statistical analysis. The significance level was set at 5%.

## Results

### Patients

**Figure 2:**
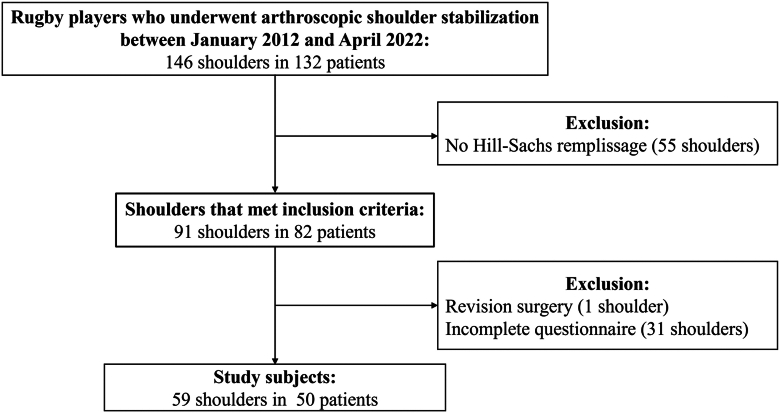
Patient flow diagram. *ROM*, range of motion.

A total of 146 shoulders in 132 rugby players underwent arthroscopic shoulder stabilization, and 91 shoulders in 82 patients met the inclusion criteria (Fig. 2). Thirty-two shoulders were excluded: revision surgery, one shoulder; incomplete questionnaire, 31 shoulders. Thus, the remaining 59 shoulders in 50 patients were included in this study. The patients consisted of 48 males and 2 females, with a mean age at surgery of 19 years (range, 14-36). The mean follow-up was 31 months (range, 13-56). The detailed characteristics of the subjects are described in Table I.

**Table I:** Patient demographic.

	Data
No. of shoulders	59
Sex, n (%)	
Male	48 (96)
Female	2 (4)
Age at surgery, yr∗	19 (14-36)
Affected shoulder, n (%)	
Right	31 (53)
Left	28 (47)
No. of dislocation/subluxation∗	6 (1-30)
Time from first dislocation to surgery, mo∗	4 (1-18)
Position, n (%)	
Forwards	27 (54)
Backs	23 (46)
Category, n (%)	
Professional/international	6 (12)
Local/regional	3 (6)
College league	12 (24)
Junior/senior high school	29 (58)

∗ The values are presented as mean (range).

### Sports return and recovery metrics

**Figure 3:**
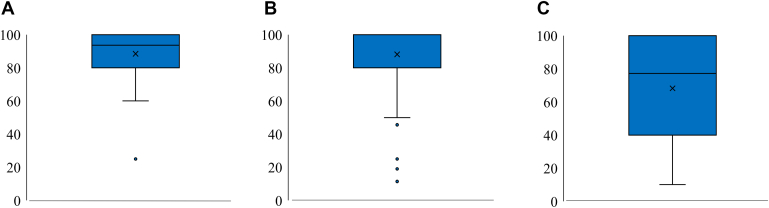
Box plot for visual analog scale for athletic performance (**A**), pain (**B**), and fear of contact (**C**).

The mean time to return to contact practice was 7 ± 5 months (range, 3-36), and the mean time to RTP was 8 ± 5 months (range, 4-36). Four shoulders (7%) experienced reinjury, and no shoulders underwent revision surgery. Three patients with recurrence returned to rugby after physiotherapy; however, one patient who did not receive sufficient physiotherapy was unable to return to the sport. Overall, 49 athletes (98%) returned to their preinjury sports level. The median of VAS values was as follows: 91 for athletic performance (range, 25-100), 100 for pain (range, 10-100), and 70 for fear of contact (range, 10-100) (Fig. 3). The median of the sum of the 3 VAS values (athletic performance, pain, and fear of contact) was 261 (range, 90-300).

### Relationship between ranges of motion and visual analog scale values

Seven shoulders were excluded from the 59 shoulders: six shoulders due to a lack of ROM data and one shoulder due to a failed sports return. The relationships between ROMs and the VAS values were analyzed in the remaining 52 shoulders. The ROM data are presented in Table II.

**Table II:** Pre- and postoperative range of motion.

	Preoperative	Postoperative 3 mo	Postoperative 6 mo	Postoperative 12 mo
AE	165 ± 13 (130-180)	158 ± 13 (110-180)	166 ± 8 (140-180)	171 ± 6 (150-180)
ER1	55 ± 16 (20-90)	40 ± 16 (5-70)	50 ± 15 (15-80)	55 ± 15 (20-80)
ER2	81 ± 16 (30-115)	65 ± 15 (30-95)	73 ± 12 (50-100)	76 ± 11 (50-90)

*AE*, anterior elevation; *ER1*, external rotation with the arm at the side; *ER2*, external rotation in 90-degree abduction.

The values are presented as mean ± standard deviation (range).

The single regression analysis revealed that the total VAS value had a significant relationship with AE (ß: 0.33, *P* = .018) and ER1 (ß: 0.35, *P* = .010) at 3 months after surgery (Table III). The multiple regression analysis also demonstrated that the total VAS value had a significant relationship with AE (adjusted ß: 0.36, *P* = .015) and ER1 (adjusted ß: 0.49, *P* = .002) at 3 months after surgery. The post hoc power analysis indicated that the statistical power was 0.65 for AE at 3 months and 0.87 for ER1 at 3 months.

**Table III:** Regression analysis of visual analog scale score for fear of contact.

	Model 1∗		Model 2†	
	ß	*P* value	Adjusted ß	*P* value
Age	0.03	.84	-	-
Follow-up period	0.11	.42	-	-
Preoperative period	0.06	.66	-	-
Number of dislocations/subluxations	−0.04	.77	-	-
AE				
Preoperative	0.11	.45	0.10	.52
Postoperative 3 mo	0.33	.018	0.36	.015
Postoperative 6 mo	−0.01	.95	−0.02	.89
Postoperative 12 mo	−0.03	.87	−0.01	.95
ER1				
Preoperative	0.21	.13	0.23	.12
Postoperative 3 mo	0.35	.010	0.49	.002
Postoperative 6 mo	0.09	.56	0.14	.42
Postoperative 12 mo	0.15	.36	0.21	.30
ER2				
Preoperative	0.09	.54	0.09	.59
Postoperative 3 mo	0.24	.085	0.28	.065
Postoperative 6 mo	0.15	.32	0.19	.26
Postoperative 12 mo	−0.04	.83	−0.03	.88

*AE*, anterior elevation; *ER1*, external rotation with the arm at the side; *ER2*, external rotation in 90-degree abduction.

∗ Simple regression analysis.

† Multiple regression analysis: adjusted by inputting age, questionnaire collection time, preoperative period, and number of dislocations/subluxations as covariates.

## Discussion

This study demonstrated that arthroscopic Bankart repair with augmentations for traumatic anterior shoulder instability in rugby players yielded a 98% RTP rate, with return to contact practice at seven months and participation in practice games at eight months postoperatively. Most patients showed favorable recovery in terms of performance and pain, while those with fear of contact demonstrated greater variability in VAS values. The total VAS values were significantly associated with AE and ER1 at three months after surgery, supporting our hypotheses.

This study demonstrated an excellent 98% RTP rate within eight months postoperatively, although the time to return to practice games was longer than that reported in previous studies.^10^^,^^14^^,^^20^^,^^22^^,^^24^^,^^26^^,^^29^^,^^38^ Three out of 4 recurrent cases returned to the sport without revision surgery. Our surgical techniques with selective augmentations and postoperative rehabilitation may have contributed to the excellent RTP, even in a contact sport like rugby.^10^

In this study, an average of seven months was required to resume contact activities, even though the protocol allowed return at four months postoperatively. While contact activities are generally allowed between 4 and six months postoperatively, the return is often delayed.^8^^,^^21^ This delay is associated with multiple factors, including limited ROM, insufficient strength, prolonged shoulder pain or discomfort, and psychological issues.^8^^,^^21^^,^^22^ Therefore, the proper timing for resuming contact activities should be determined on a case-by-case basis, taking into consideration the functional and psychological status of each patient.

The influence of psychological factors, including fear of contact, on rugby players' performance during their RTP is not well understood. Previous studies have shown that psychological aspects significantly impact the level of success of athletes' RTP.^4^^,^^11^^,^^25^^,^^31^ In this study, 98% of athletes returned to their preinjury level with good recovery of performance and pain; however, some patients had a fear of contact even after successful return to the sport. A previous study has indicated that anxiety about the recovery status is one of the reasons for being unable to RTP with full performance after surgery.^25^ Therefore, it may be important for therapists to understand patients' concerns and frequently inform patients about their current and expected athletic condition.

This study demonstrated a significant association between ROM at postoperative three months and the total VAS values. Previous studies have suggested that muscle strength and joint position sense are restored to the preinjury level between 3 and 6 months after surgery, which are closely associated with ROM.^1^^,^^33^^,^^36^^,^^37^ Moreover, ROMs at postoperative three months are reported to better reflect early capsular mobility, pain control, and rehabilitation progress than at six months.^7^^,^^12^^,^^16^^,^^19^^,^^32^ The delayed recovery of ROMs may be associated with limited athletic performance even after return to a game due to incomplete restoration of muscle strength, proprioception, and capsular mobility. Early attainment of better ROMs may be important for rugby players after shoulder stabilization to support return to sport with higher athletic performance.

The results of this study have several implications. First, early intervention for psychological factors may be required. Previous research has reported that psychological approaches can enhance recovery both physically and psychologically.^13^^,^^23^ Furthermore, psychological readiness has been recognized as a critical factor influencing the success of RTP.^13^^,^^23^ Second, early ROM attainment, followed by progressive contact drills, may be crucial for better RTP. These approaches may improve athletes' confidence and decrease the risk of reinjury.^6^^,^^9^ Therefore, a rehabilitation protocol that simultaneously addresses psychological and physical aspects may contribute to better RTP.

This study has several limitations. First, this was a retrospective study using a subjective questionnaire. This may cause a recall bias. Standardized psychological assessment tools, such as the Tampa Scale of Kinesiophobia may be more appropriate. Second, the rate of incomplete questionnaires was high. This may lead to a selection bias. Third, the sample size was not large enough, as indicated by the post hoc power analysis. Fourth, we only evaluated ROM as a clinical assessment. Regardless of these limitations, we believe that this study provides important insight into the treatment of rugby players who require shoulder stabilization.

## Conclusion

Arthroscopic shoulder stabilization resulted in a 98% RTP rate in rugby players. The VAS score for fear of contact was relatively low, even in patients who achieved complete return. The total VAS value was correlated with ROM at three months postoperatively. A rehabilitation protocol that simultaneously addresses psychological and physical aspects may be required for better RTP.

## Acknowledgments

The authors are grateful to Koharu Matsuki for English language editing.

## Disclaimers

Funding: No funding was disclosed by the authors.

Conflicts of interest: Norimasa Takahashi has received speaker honoraria from DePuy Synthes, Medacta International, outside the submitted work. Hiroyuki Sugaya has received speaker honoraria from DePuy Synthes, Smith & Nephew, Zimmer, Biomet, and stryker, outside the submitted work. The other authors, their immediate families, and any research foundation with which they are affiliated have not received any financial payments or other benefits from any commercial entity related to the subject of this article.
